# The Antifouling Mechanism and Efficacy of Graphene Nanomaterials in Composite Coatings against Marine Diatoms

**DOI:** 10.1021/acsomega.5c09053

**Published:** 2025-11-25

**Authors:** Michael R. Kelly, Andreas Erbe, Ingrid G. Hallsteinsen, Hilde L. Lein

**Affiliations:** Department of Materials Science and Engineering, 8018 NTNU, Sem Saelands Vei 12, Trondheim 7034, Norway

## Abstract

The urgent need for sustainable antifouling solutions in marine environments has intensified the search for alternatives to toxic biocides. One promising approach involves embedding graphene nanomaterials into polymer composites. While graphene’s antifouling properties have been extensively studied in solution, its mechanisms within solid composites remain unclear—particularly whether its effects are primarily chemical, such as oxidative stress, or physical, such as mechanical disruption. This study investigates the antifouling mechanisms of native, unmodified graphene and graphene oxide embedded in epoxy composite coatings targeting marine diatoms under laboratory conditions. Both graphene-based coatings significantly outperformed pure epoxy in reducing diatom adhesion in flow-through systems, using both monocultures and mixed algal cultures, with efficacy increasing alongside filler concentration. A comprehensive suite of characterizations—including surface energy analysis, reactive oxygen species (ROS) measurements, and scanning and transmission electron microscopy—was employed to elucidate the mode of action. Unlike commercial antifouling coatings, the high surface energy of these composites rules out fouling release as the dominant mechanism. ROS measurements indicated minimal oxidative stress, suggesting that chemical toxicity is not the primary driver. Microscopy revealed membrane disruption as the main cause of cell death, primarily through contact-mediated phospholipid damage. Furthermore, cellular assays showed higher cell mortality on graphene-containing surfaces compared with those with graphene oxide, reinforcing the role of mechanical disruption. Overall, these findings demonstrate that graphene nanomaterials confer antifouling activity primarily through direct contact interactions, highlighting their potential for durable, nontoxic marine coatings. However, to fully leverage the biocidal properties of graphene and graphene oxide, efficient removal of dead foulants remains a critical challenge.

## Graphical abstract

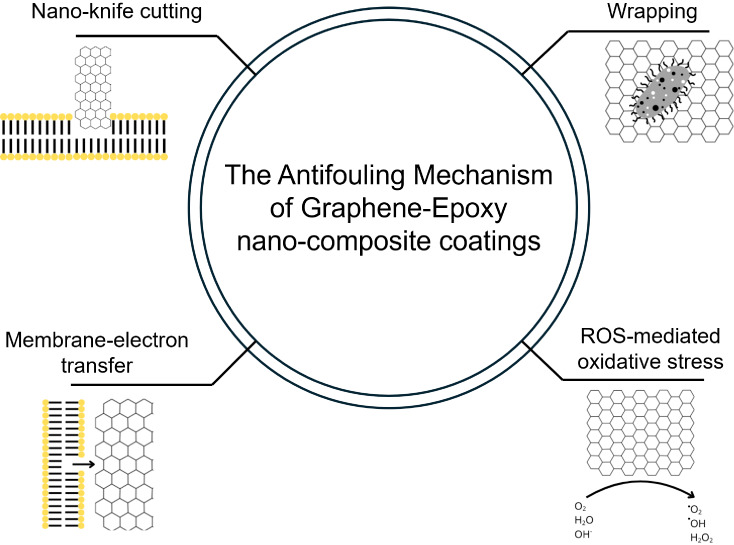

## Introduction

Marine organisms can accumulate on surfaces, leading to a phenomenon known as biofouling.^1,2^ This accumulation negatively affects the hydrodynamic volume and friction of marine vessels, resulting in increased drag, reduced speed, and higher fuel consumption,^3,4^ and it can cause structural issues in marine aquaculture.^5^ To combat this issue, applying antibiofilm coatings to these surfaces is a common solution. Self-polishing coatings containing biocides, for instance, tributyltin (TBT) or copper, are effective in preventing fouling.^6–8^ However, due to their significant toxicity to nontarget species, these substances have been banned or increasingly restricted.^9–14^

These restrictions highlight the need for environmentally friendly alternatives in the field of antifouling coatings, and in response, significant advancements have been made in developing new solutions. Among them are coatings based on biomimetic microstructures,^15–17^ fouling release coatings,^18–20^ self-assembled monolayers,^21,22^ zwitterionic coatings,^23,24^ and slippery liquid-infused porous structures,^25^ though these coatings are all limited in several ways, such as complexity in design, durability, specificity to certain fouling species only, and scalability issues. There have been solutions based on polymer–nanoparticle composites suggested, as well. Less harmful biocides, such as silver and some metal oxides in self-polishing coatings, have been investigated.^26^ However, these have limited durability due to the coating eventually being fully depleted. Recently, conductive coatings including carbon nanotubes^27^ and composites^28^ have been investigated. Though these provide an innovative and environmentally friendly solution, their complexity and technical requirements limit their wide-scale application.^29^ Photocatalytic coatings use light-activated substances to degrade fouling organisms.^30,31^ Although these provide excellent antifouling properties and are environmentally friendly, they require strong UV exposure, which may be limited, for instance, to the bottom of shipping hulls.

There has also been an increase in studies focused on graphene (G) nanomaterials as potential solutions for antibiofilm coatings. G is a 2D nanomaterial comprising sp^2^-bonded carbons arranged in a hexagonal lattice.^32^ The unique structure of 2D nanomaterials can result in extraordinary physicochemical properties, enabling technical solutions to address global challenges in medicine, water scarcity, and energy production.^33^ G-based nanomaterials, in particular, have attracted substantial research interest over the past decade because of their exceptional mechanical, electronic, and thermal properties.^34^ Decorating carbon nanotubes or tungsten nanorods with graphene nanomaterials has shown to effectively prevent fouling due to their superhydrophobic properties.^35,36^ Recently developed epoxy-G composites have shown encouraging anticorrosive properties.^37,38^ There have been a considerable number of reports on the cytotoxicity and antimicrobial effect of G, graphene oxide (GO) nanocomposites, and derivatives.^39–42^ Also, several groups have shown that decorating nanoparticles on the surface of GO further improves its antimicrobial effect.^43,44^ However, the mechanism by which pure G and GO act as antimicrobial agents remains controversial and continues to be a subject of debate.

Generally, it is believed that the antimicrobial action of G and GO nanosheets is due to both physical and chemical factors.^42,45^ A purely chemical factor involves the overproduction of reactive oxygen species (ROS). ROS has been found to oxidize fatty acids leading to the production of lipid peroxides that stimulate a chain reaction, eventually leading to the disintegration of the cell membrane followed by cell death.^46^ The edges of the sheets could serve as active sites for redox reactions due to the abundance of edge-bound defect sites.^47–50^ A physical factor is a “nano-knife” effect, which involves graphene sheets penetrating the cell membrane upon contact. The penetration is driven by strong dispersion interactions between G and the lipid molecules. When the membrane is broken, a leakage of intracellular components such as RNA, DNA, phospholipids, and proteins will occur, resulting in cell death.^40^ Modeling results suggest that the nanosheets readily penetrate cell membranes when interacting with cells in an orthogonal orientation; at this orientation, the sharp edges possess the lowest energy barrier to initiate local penetration through the lipid bilayer.^51^ The size and orientation of the G sheets influence their ability to pierce cell membranes, with smaller sheets more easily penetrating the lipid bilayer, causing electron transfer-mediated oxidative stress, while larger sheets tend to lie flat on the cell membrane surface, and induce structural changes caused by the strong pulling forces from the nanosheets on phospholipid molecules, leading to oxidative stress-like effects that impair membrane integrity.^52,53^ A second, purely physical, mechanism reported is a wrapping mechanism of larger sheets preventing bacterial proliferation.^54^

In addition, several combinations of mechanical and chemical factors have been proposed. The mechanical penetration discussed above can act as a direct electron conduit across the membrane. Electron transfer from the penetration has been found to modulate intracellular redox states and cause oxidative stress, independent of ROS, also leading to membrane disruption.^53,55,56^ However, others have reported that the antibacterial activity is caused by electron transfer from the bacterial membrane to the G and GO surface,^57^ granted that the G and GO are on a conductive substrate. When in contact with bacteria, graphene acts as an electron acceptor, drawing electrons away from the bacterial membrane, which creates the oxidative stress.^57^ They suggest that the surface of the nanosheet, rather than the edges, is chiefly responsible for antimicrobial activity.^58^

The antibiofilm effectiveness of G-polymer composite coatings remains unclear.^59^ Most research on the cytotoxicity and antimicrobial properties of G-nanomaterials has focused on materials in solution rather than those confined within composite coatings, such as Mejías Carpio et al.,^60^ showing the cell encapsulation mechanism and Kang et al.,^61,62^ showing G sheet membrane penetration in solution. When G-nanomaterials are embedded in a polymer matrix, their availability and orientation may change significantly, which could in turn alter their biocidal properties.

To the best of our knowledge, limited research has been done on the antifouling efficacy and mechanisms of native and unmodified graphene and graphene oxide in epoxy nanocomposite coatings. This work investigates the antifouling efficacy and underlying mechanisms of G-epoxy nanocomposite coatings against marine diatoms. The coatings were fabricated by incorporating G and GO into an epoxy matrix and tested for biofilm resistance under controlled marine conditions. Antifouling performance was quantified through diatom adhesion studies over extended exposure periods. To elucidate potential biocidal mechanisms, we evaluated surface physicochemical properties, ROS generation, and the structural state of G within the composite. Cellular investigations using electron and fluorescence microscopy were conducted to examine the membrane integrity and cell viability. These complementary analyses aim to determine whether G’s antifouling effect in a polymer-bound state is driven by chemical mechanisms, such as oxidative stress, or by physical interactions, such as mechanical disruption via sharp nanosheet edges. We show here that G- and GO-based epoxy coatings demonstrated a strong antifouling performance by significantly reducing diatom adhesion, primarily through contact-induced membrane disruption.

## Materials and Methods

### Materials and Synthesis

Epikote 828 resin, 0.6 wt % G-Epikote dispersion, and 10 wt % GO paste were acquired from CealTech AS (Stavanger, Norway). Poly­(propylene glycol) bis­(2-aminopropyl ether) (Jeffamine D230) curing agent was purchased from Sigma-Aldrich (Saint Louis, USA). Acetone (100%) and Ethanol (96%) were purchased from Merck Life Sciences NV (Amsterdam, Netherlands). Sandblasted high-density polyethylene (PE) substrates (12.6 mm diameter, 4 mm thickness) were provided by the NTNU Workshop (Trondheim, Norway).

Equal parts of Epikote 828 epoxy resin and acetone were mixed into an epoxy solution. For the G nanocomposites, the G-Epikote 828 dispersion was mixed with pure Epikote 828 epoxy resin and acetone to achieve final G concentrations of 0.500 wt % in the polymer after solvent evaporation. For the GO nanocomposites, the GO paste was added to Epikote 828 resin, and then a solvent of 50/50 acetone and ethanol was added such that there were equal parts solvent and epoxy resin, with 0.500 wt % GO in the polymer after solvent evaporation. Before deposition, JD230 curing agent was added to the epoxy sols and the nanocomposite slurries to a concentration of 20 wt % curing agent to epoxy.

The spray deposition of the slurries onto polyethylene (PE) substrates was done using an Airbrush paint gun (Art. 17–371, Biltema, Linköping, Sweden) with a 0.3 mm nozzle with a nitrogen gas pressure of 2.0 bar. Before the deposition, the substrates were sonicated in ethanol for 5 min to sterilize and clean the surface and then subsequently dried in a fume hood. The slurries were sonicated for 5 min to redisperse any precipitated particles. The paint gun was held at approximately 10 cm distance from the sample during the deposition. The samples were left in the fume hood overnight in order for the solvent to evaporate, and then the process was repeated for a total of three applied layers. The samples were then put into a Carbolite Gero PF sol–gel oven (Hope Valley, England, UK) at 60 °C for 4 h to complete the curing process, with a final dry film thickness of approximately 400 μm. The samples were kept in sterile sample trays.

Coumarin-3-carboxylic acid (3CCA), phosphate buffer (1M, pH 7.4), and hydrogen peroxide (30%) were purchased from Merck Life Sciences NV (Amsterdam, Netherlands). SYTOX Green Nucleic Acid Stain (5 mM solution in dimethyl sulfoxide (DMSO)) was purchased from Thermo Fisher Scientific (Massachusetts, USA). Hexamethyldisilazane (HMDS) (≤99%) and glutaraldehyde (50% in H_2_O) were purchased from Sigma-Aldrich (Saint Louis, USA), D­(+)-saccharose (sucrose) (powder, 100%) was purchased from VWR Chemicals (Pennsylvania, USA), and PHEM buffer (5×) was prepared by the Cellular & Molecular Imaging Core Facility (CMIC) group at St. Olavs Hospital (Trondheim, Norway) following the method by Montanaro et al.^63^

The mixed diatom cell culture was provided by the NTNU SeaLab (Trondheim, Norway). Conwy nutritional medium and Silicate nutritional solution (Na_2_SiO_3_·5H_2_O), used as growth medium, were also provided by NTNU SeaLab. *Phaeodactylum tricornutum* monoculture was purchased from the Culture Collection of Algae and Protozoa (CCAP) Biological Resource Centre within the Scottish Association for Marine Science (SAMS) (Dunbeg, Scotland). Algae suspensions were grown in autoclaved and filtered seawater with additions of the growth medium.

### Biological Characterization

Antifouling efficacy of the epoxy and epoxy nanocomposite samples was investigated using a bioreactor, as shown in Figure 1. The bioreactor was prepared by the NTNU Workshop.

**Figure 1:**
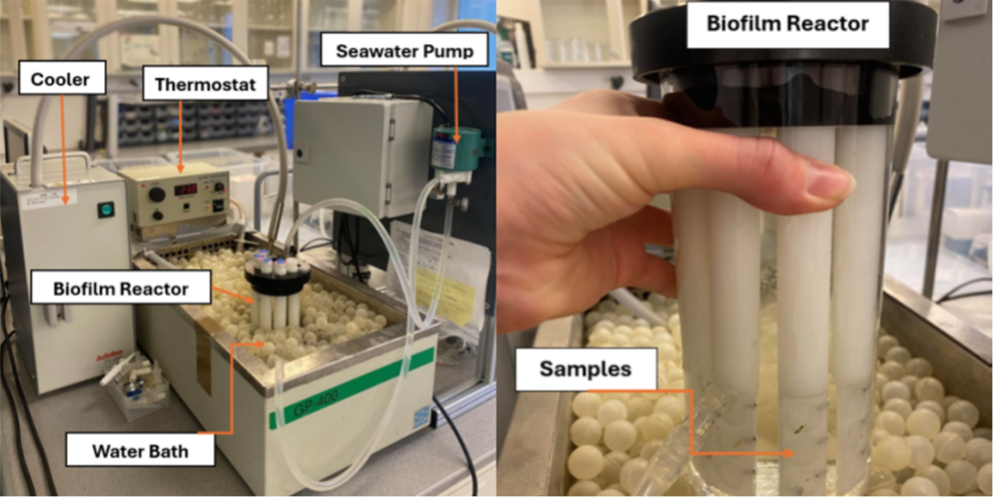
Experimental setup for the biofilm reactor experiment for antifouling efficacy testing. The image includes the bioreactor along with the water bath, cooling, and heating system, as well as the seawater pump. Reproduced from Kelly et al., Laboratory and Field Evaluation of Graphene Oxide and Silver Nanoparticle-Enhanced Synergistic Silicone Fouling Release and Biocidal Coatings for Marine Antifouling.^64^

700 mL of seawater containing algae culture was pumped through the bioreactor, with samples mounted on the rods submerged in the bioreactor, which was kept at 10 °C and 4 °C for the mixed culture and the *Phaeodactylum tricornutum* culture, respectively. Pure PE substrates were used as control samples. The seawater pump was connected to the bioreactor through plastic tubes with a pump speed of 1 L/min. The experiment was run for 3 weeks for the mixed algae culture and 10 days for the *Phaeodactylum tricornutum* algae culture, in order for the respective cultures to reach maturity. The samples were subsequently removed and gently rinsed in deionized water to remove salt particles and left to dry in a fume hood overnight before imaging. Triplets of each sample were tested. The exposure tests were run three times. For investigating the biofilm formation over time against *Phaeodactylum tricornutum*, an experiment was run for a duration of 1 week, with samples being removed after 1, 4, and 7 days. The antifouling efficacy of the samples was quantified by manual counting of cells adhered to the surface with the use of optical imaging using an Alicona Infinite Focus SL optical microscope with a 50× magnification lens. A set of ten images was taken on each sample, and the diatom growth was expressed as the density of diatoms on a total area of 0.166 mm^2^.

Fluorescence imaging was employed to inspect cells on the surface, and a staining solution was used to differentiate the live cells from the dead cells. SYTOX Green Nucleic Acid Stain was used to visualize the dead cells, since this stain cannot enter the cells of live bacteria with an intact membrane^65^ but will stain the DNA in the nucleus of dead cells with compromised cell membranes. The autofluorescence of chlorophyll A was used to visualize the live cells. The 5 mM SYTOX Green stain was diluted with deionized water to a working solution of 167 nM. Epoxy and epoxy nanocomposite samples were submerged in a solution of *Phaeodactylum tricornutum* algae culture overnight. They were then gently rinsed with deionized water and subsequently submerged in the SYTOX Green staining solution for 30 min with no light exposure and incubated at room temperature for 30 min. The staining solution was discarded, and the samples were gently rinsed with deionized water before imaging with a Zeiss 700 confocal laser scanning microscope (Oberkochen, Germany). The SYTOX Green stain has an excitation and emission maximum of 504 and 523 nm, respectively; a 488 nm argon-ion laser was used for excitation with an EGFP filter cube. A 639 nm laser source was used to excite chlorophyll A with a BP filter of 650 nm to 700 nm. An EC Plan Neofluar objective with 10× magnification was used with air immersion, with a pinhole of 1 Airy Unit.

Chemical fixation of *Phaeodactylum tricornutum* cells on the sample surfaces was done as a series of dehydration and drying steps after fixation in glutaraldehyde. The fixation was done in a solution of 2.5% glutaraldehyde, 1.5× marPHEM buffer with 9% sucrose, by dilution of stock solutions of glutaraldehyde, PHEM buffer, and sucrose powder with deionized water. Volumes of 0.5 mL fixative were added to the wells of 24-well plates along with the coated samples (after incubation in a *Phaeodactylum tricornutum* culture for 24 h at room temperature) and left in a fridge at 4 °C overnight. A series of dehydration steps with 25%, 50%, 75%, and 100% ethanol was done with 10 min between each step, followed by a drying process in 1:2, 1:1, and 2:1 (HMDS: ethanol) mixtures before three repetitions of drying with 100% HMDS. The samples were left to dry at room temperature overnight.

The samples with fixed cells were mounted on stubs with carbon tape and sputter-coated with gold nanoparticles (80 mA, 15 s) with an Edwards Vacuum Sputter Coater S150B, and then imaged using a Hitachi S-3400 N scanning electron microscope (SEM) with 5 kV accelerating voltage and a secondary electron detector.

### Material and Surface Characterization

Transmission electron microscope (TEM) lamellae were prepared in an FEI Helios NanoLab DualBeam focused ion beam (FIB) instrument (Massachusetts, USA). The samples were loaded in a JEOL 2100F TEM instrument (Tokyo, Japan) and measured in scanning electron diffraction (SED) mode. The diffraction images were analyzed to identify the diffraction pattern in each image, and the diffraction intensity was mapped for each sample.

3CCA was used as a fluorescent probe molecule for the indirect quantification of hydroxyl radical generation, based on methods developed by Manevich et al.^66^ A 5 mM stock solution of 3CCA was prepared in 20 mM phosphate buffer at room temperature, and it was stored without light exposure at 4 °C. Working solutions of 2 mM 3CCA were made by diluting the stock solution with deionized water, and coated samples were placed in the solution and left for 1, 4, and 7 days. Control samples with 1% hydrogen peroxide were also made, and a serial dilution of hydrogen peroxide was also prepared. Aliquots of 150 μL were drawn, and fluorescence was measured using the Molecular Devices SpectraMax i3x Microplate Reader (San Jose, California, USA), with excitation and emission wavelengths of 395 and 450 nm, respectively. The background signal from a blank sample of water was subtracted from all emission spectra.

Contact angle measurements were made using the sessile drop technique with a Krüss DSA100 Drop Shape Analyzer (Hamburg, Germany) and the Krüss ADVANCE software for measuring the contact angles. Water was used as the liquid. The contact angles were averaged over 3–5 parallel measurements at different positions on the surface. The Young–Laplace method was used as the fitting method for the measurements. Diiodomethane was also used to obtain contact angle measurements of a dispersive liquid as well. Contact angle measurements were subsequently converted to surface free energy using the OWRK method.^67,68^ Water was chosen as the polar liquid, and diiodomethane was chosen as the dispersive liquid.

All experiments were performed with three independent replicates, unless otherwise stated. Quantitative data are reported as the mean ± standard deviation. Data analysis and plotting were carried out in Python by using the numpy and matplotlib.pyplot libraries. No formal hypothesis testing was applied; comparisons between groups are based on observed trends in the measured values.

## Results

### Antifouling Efficacy

The number of algae attached to the surfaces of the epoxy, G, and GO nanocomposite coatings after exposure in the bioreactor was quantified, and the density of the cells is shown in Figure 2. The surfaces were exposed to both a diatom-dominated mixed algae culture for 3 weeks and a *Phaeodactylum tricornutum* algae culture for 10 days in the bioreactor. These are compared to those of uncoated PE substrates.

**Figure 2:**
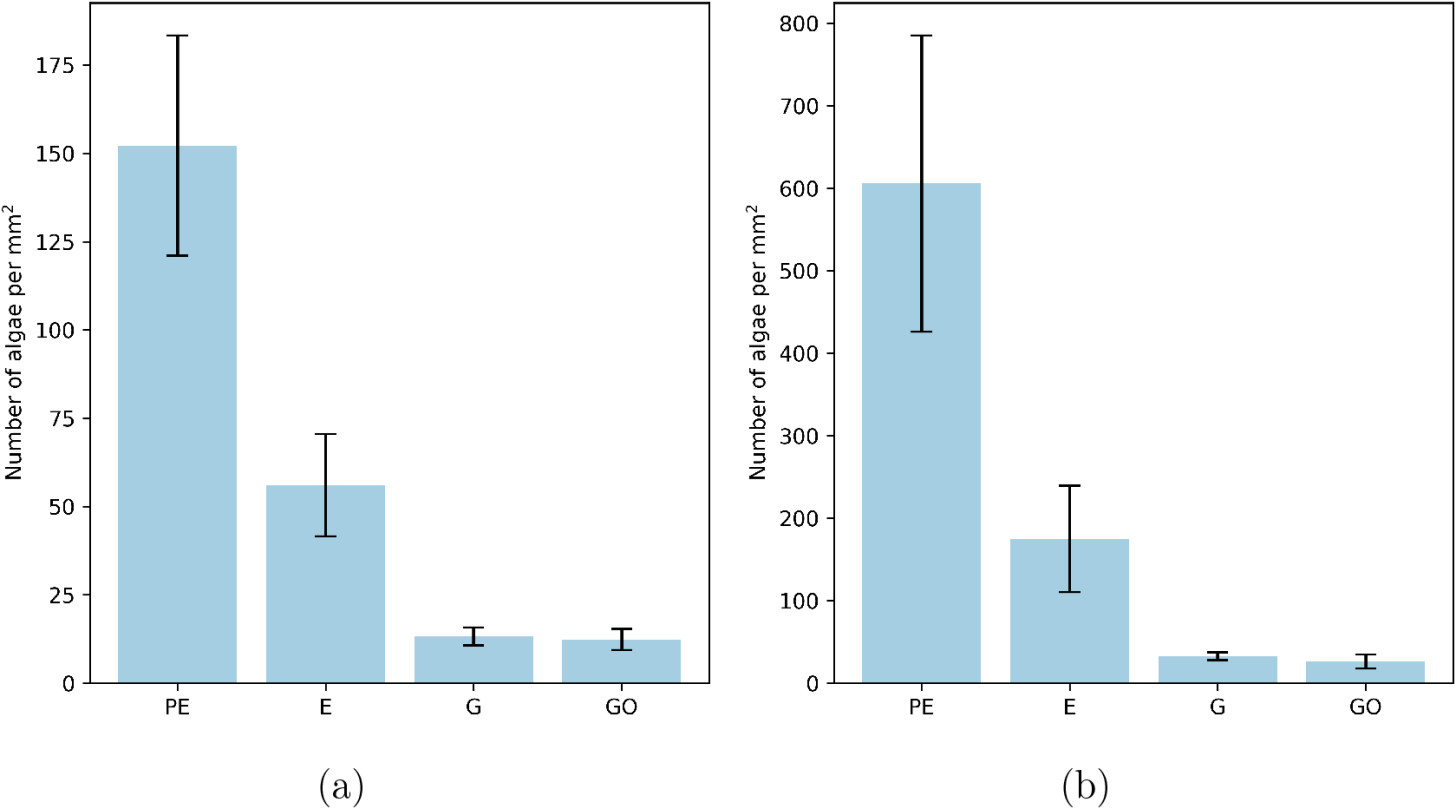
Number of algal cells per mm^2^ on the surfaces of uncoated polyethylene (PE) substrates, epoxy (E) coating, and 0.500 wt % G and GO after exposure to (a) a mixed algae culture after 3 weeks in the bioreactor and (b) a *Phaeodactylum tricornutum* monoculture after 10 days in the bioreactor. Data represent the mean ± standard deviation from three independent replicates.

The amount of algae attached to the surfaces of uncoated PE is significantly higher than that for the coated surfaces, both with and without the G nanomaterial present. The density of cells attached to the pure epoxy-coated samples is significantly reduced in comparison to the untreated surface, to just 37% and 29% relative to the PE control for the mixed culture and monoculture, respectively. This is likely due to them having smoother surfaces than the PE substrate. Adding G and GO significantly increased the AF capabilities further. The algal density on G was just 9% and 5% relative to the PE control for the mixed culture and monoculture, respectively. The algal density on GO was just 8% and 4% relative to the PE control for the mixed culture and the monoculture, respectively. The nanocomposite materials show significant antifouling capabilities when exposed to both the monoculture and mixed algae culture, showing the ability to target several fouling species. No measurable difference in the antifouling capabilities was found between the G and the GO coatings. The algal density of the *Phaeodactylum tricornutum* monoculture was significantly higher than for the mixed diatom culture, stemming from differences in growth conditions of the different cultures before sample exposure, and direct comparisons of cell density are not meaningful between the different experiments; only differences between each sample within the same exposure test are compared. The composite samples have the same AF efficacy against the mixed diatom culture and the *Phaeodactylum tricornutum* monoculture; for the remaining AF and cellular investigations, we consider only the monoculture and assume that the mechanisms are the same.

In order to elucidate the AF performance of the fillers over time, the number of cells on the uncoated PE substrates and substrates coated with epoxy, G, and GO after 1, 4, and 7 days of exposure to *Phaeodactylum tricornutum* in the bioreactor were quantified and compared. The percentage of attached cells per mm^2^ on the coated surfaces relative to that on the uncoated PE substrates is shown in Table 1.

**Table 1:** Percentage of Cell Attachment on to the Surfaces Coated with Epoxy, 0.500 wt % G, and 0.500 wt % GO Relative to Uncoated PE Substrates after 1, 4, and 7 Days of Exposure to *Phaeodactylum tricornutum* in the Bioreactor^a^

	1 Day	4 Days	7 Days
Epoxy	10%	21%	36%
G	11%	5%	6%
GO	10%	4%	4%

a Percentage of cell attachment = cells on coated sample after *x* days/cells on uncoated PE substrate after x days × 100%; *x* = 1, 4, 7.

There is a nearly linear increase in the surface coverage on the epoxy-coated surface from 10% to 36%, implying a loss of antifouling effect. The 0.500 wt % G- and GO-coated surfaces had a similar trend, with a decrease in the relative attachment of cells from 10% to 11% down to just 5% to 6% after 4 and 7 days, respectively; after 1 day, cell attachment on the G and GO coatings was comparable to that on epoxy, but after 4 days it was reduced by approximately 76% and 81%, respectively, and after 7 days by 83% and 89%, relative to the epoxy surface. This indicates that G and GO work in a similar fashion, where an initial settlement of cells is seen after a day; then, the presence of the G and GO nanofillers prevents further cell attachment and effectively reduces the fouling relative to uncoated surfaces. This could be due to G and GO killing the cells, which then are only loosely held to the surface.

The surface energy of the epoxy and nanocomposite coatings was determined from contact angle measurements by use of the OWRK method, and these results can be found in the Supporting Information. The surface energies of the epoxy and G coatings were approximately 60 mN/m, with the GO surface slightly higher at just above 70 mN/m. All coatings had significantly higher surface energies than 20 mN/m to 30 mN/m, the region for which bacterial adhesion is minimal and the fouling release mechanism is present;^69,70^ none of the coated samples have a fouling release mechanism, and thus any improvement in their antifouling efficacy must be a result of a different mechanism. Investigations into the coating integrity and degradation after environmental exposure were not done, as this is outside the scope of the work.

### Reactive Oxygen Species Generation

To check for the chemical mechanism, where the fillers would generate ROS, we use an indirect measurement of ROS generated via the fluorescent measurements of the hydroxylated 3CCA probe molecules as shown in Figure 3.

**Figure 3:**
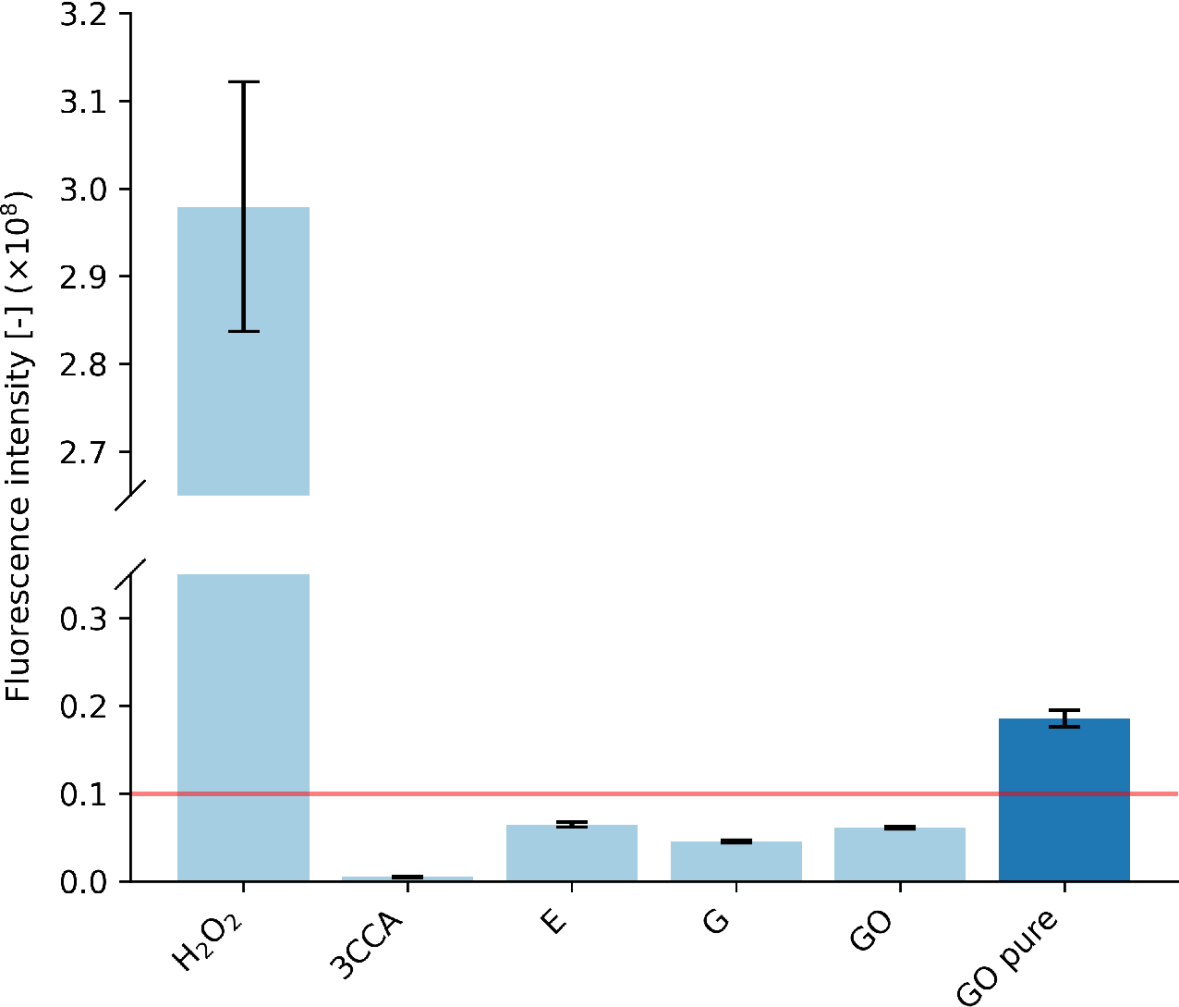
Fluorescent intensity of the 7-hydroxycoumarin-3-carboxylic acid probe molecule in samples exposed to the epoxy (E), and G and GO composite surfaces for 1 week as well as 1% hydrogen peroxide and a solution with 3CCA as controls. GO pure = pure GO paste. Data represent mean ± standard deviation from three independent replicates. The red line indicates the sensitivity of the measurement.

The control sample exposed to 1% hydrogen peroxide shows a fluorescent intensity significantly higher than that of all other samples. The 3CCA control sample shows the least fluorescence intensity, while the solutions exposed to the epoxy, G, and GO nanocomposites show slightly higher values. A solution with pure G was not tested, as the G was provided only in an epoxy-G dispersion and was unavailable as pure G. The solution exposed to pure GO showed a significant increase in the fluorescent signal in comparison with the 3CCA control and the epoxy, G, and GO coatings. The low fluorescence intensities imply that the amount of ROS generated by the epoxy, G, and GO coatings is very low.

To investigate the sensitivity of the method, a serial dilution from 1% to 0.001% hydrogen peroxide was measured, and the results are found in the Supporting Information. This experiment yielded a noise floor of approximately 0.10 × 10^8^, which is above the measurements of all coated samples. The determined level is indicated in the figure as a line. Consequently, the difference between these coatings cannot be determined within the resolution of the experiment.

However, there is a significant fluorescence intensity of pure GO. This implies that increasing the amount of filler may provide a significant increase in the amount of ROS generated. It also indicates that the cell close to a GO particle would experience higher levels of ROS, which could lead to cell death in the proximity of the GO particle.

### Structural Characterization of Coatings

To identify the possibility of mechanical mechanisms, the distribution and structural characteristics of G within the epoxy matrix were further examined by using TEM. Figure 4a shows cross-sectional images of the G-epoxy composite, revealing bright contrasts indicative of crystalline domains corresponding to the G material. These observations confirm that the G retains its crystalline structure after incorporation into the polymer matrix. While G sheets are distributed throughout the coating, they appear in small agglomerates rather than as single sheets. However, they are quite well distributed throughout the coating after curing. Figure 4b presents a higher-resolution image of localized G agglomerates in the composite. The images reveal the characteristic sheet-like structure of G, with individual layers stacked or oriented at different angles. The sizes of the sheets vary, though the larger sheets are approximately 200 nm long and 10 nm to 20 nm thick. To confirm the crystalline nature of the G within the epoxy, scanning electron diffraction patterns were obtained from regions with a high density of G sheets. Figure 4c displays a representative diffraction pattern, where the observed spacing matches that of multilayer G, confirming the structural integrity after processing. The [2–10] spacing corresponds to G sheets and the [002] spacing corresponds to multilayer G.^71,72^

**Figure 4:**
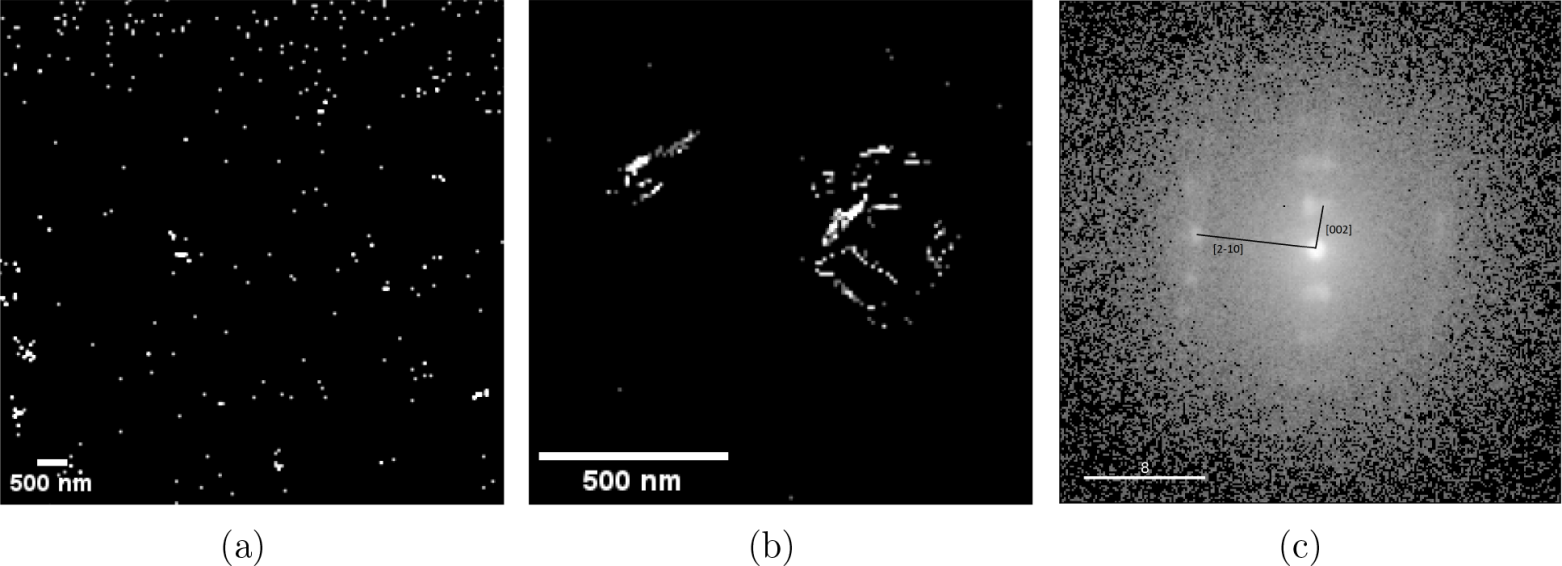
Cross-sectional TEM image of the G-epoxy composite. Bright spots indicate crystalline G domains dispersed within the epoxy matrix. The image is oriented perpendicular to the surface, with the top region representing the composite surface. (a) Overview image with lower magnification showing the distribution of crystalline domains. (b) Higher magnification image showing different orientations of G sheets within agglomerates, including parallel and perpendicular alignments. (c) Scanning electron diffraction pattern from a G-rich region, showing lattice fringes and characteristic diffraction spots corresponding to G. [2–10] spacing corresponds to G sheets and [002] spacing corresponds to multilayer G. Scale bar shows nm^–1^.

Additional diffraction patterns from various regions within the composite (shown in the Supporting Information) show similar diffraction features, suggesting that G remains crystalline throughout the matrix and that G agglomeration does not significantly disrupt the lattice structure. The different alignments of the diffraction patterns show that there is a distribution of sheet orientations in the composite. These results indicate that there are multilayer G sheets with a distribution of orientations evenly dispersed within the composite coating. The distribution of orientations suggests that some of these sheets may be oriented with the sharp edges protruding from the coating surface, implying the possibility of a mechanical cutting effect, while others may be exposed as flat sheets aligned horizontally on the surface.

### Cellular Response

In order to elucidate the AF mechanism of the fillers, the morphologies of *Phaeodactylum tricornutum* cells after exposure to the coatings were investigated. These were compared to live and healthy cells and to dead cells that were killed by intense UV irradiation. Representative SEM images of live and dead control cells are shown in Figure 5a and b, respectively. Figure 5c, d, and e shows representative cells on epoxy, G, and GO coatings, respectively, after 24 h exposure to the *Phaeodactylum tricornutum* culture.

**Figure 5:**
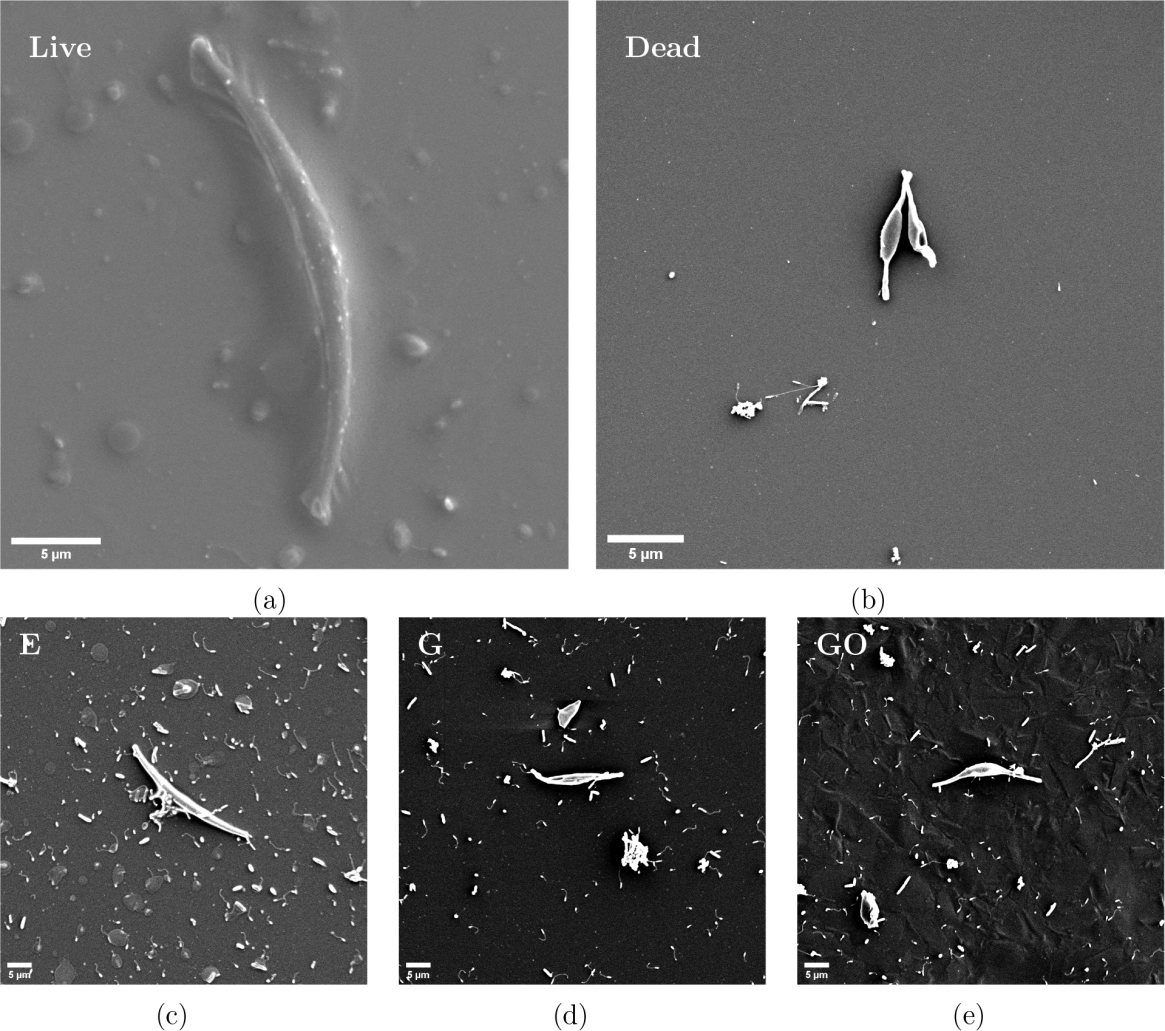
SEM images of *Phaeodactylum tricornutum*. Top row: (a) Live cell with intact fusiform morphology. (b) UV-irradiated dead cell showing collapsed structure. Bottom row: cells on nanocomposite coatings after 24 h exposure—(c) epoxy (E), (d) G, (e) GO.

Live cells exhibit typical fusiform morphology with intact frustules, showing an elongated structure with a thicker center and tapering at both ends. Dead cells appear collapsed and structurally compromised, with the ends, in particular, losing their structure. There also appears to be significant shrinkage of the cells that were UV-killed, with the cells shrinking from a length of approximately 30 μm to approximately 6 to 8 μm, or by about 4 times the length. Cells on the epoxy surface largely retained their healthy morphology, indicating that they were not killed by a disrupted membrane or ROS. Those on G-containing surfaces exhibited a higher prevalence of structural deformation, suggesting increased cell damage. This is a typical trend for these samples; in the samples with G or GO, the cells exhibit increased damage.

The cells attached to the surface of epoxy and nanocomposite coatings after 24 h of static immersion in the *Phaeodactylum tricornutum* culture were stained with SYTOX Green nucleic acid stain to visualize dead cells. The resulting images are shown in Figure 6. Both dead cells (red) and live cells (green) are shown.

**Figure 6:**
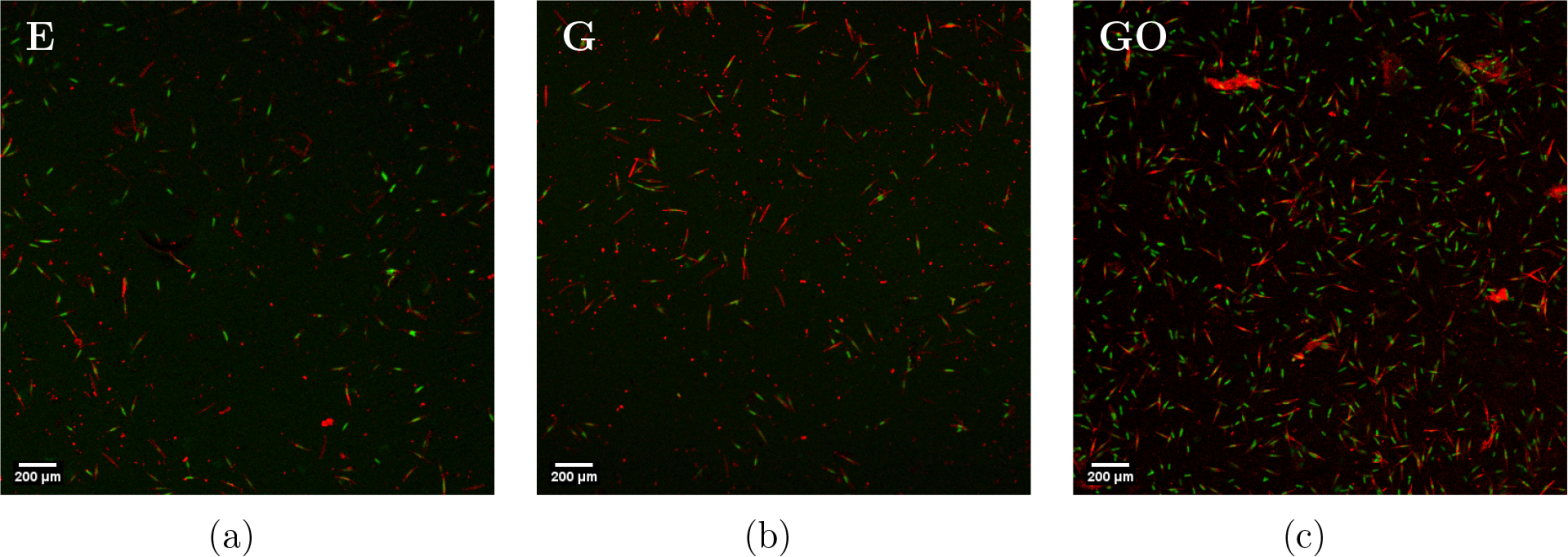
Confocal fluorescence images of *Phaeodactylum tricornutum* on (a) epoxy (E), (b) G, and (c) GO coatings after 24 h of exposure. Dead cells (red) are stained with SYTOX Green nucleic acid stain, shown alongside live cells (green).

Rough estimates of these samples showed 2.5%, 2.5%, and 6.6% surface coverage and 35%, 25%, and 35% cell viability for the epoxy, G, and GO surfaces, respectively. The dead cells cluster slightly on the surface of the epoxy coating, while the live cells spread evenly. The same clustering of dead cells happens on the G nanocomposites. There are fewer overall cells, and the ratio of the live to dead cells decreases. On the GO nanocomposite surfaces, more cells are present than on the G surface. Like with the G nanocomposite coatings, the dead cells on the GO coatings are found in larger clusters. The fluorescent images suggest that dead cells on the G and GO composite surfaces appear in larger clusters and that G coatings seem to be more efficient in killing cells than epoxy and GO coatings.

This cell viability test was done after 24 h, yet it is only after 4 days that we see any noticeable difference in the AF efficacy for G/GO in comparison to filler-free epoxy (Figure 1). Still, there is a drop in the cell viability for G surfaces. This suggests that, even though both G and GO are equally effective at preventing microfouling, G kills more cells in the initial stages, possibly indicating a difference in the AF mechanism of G and GO.

## Discussion

The mode of cellular damage caused by intense UV irradiation is comparable to that of oxidative stress, and the damage may look similar.^73^ The UV-irradiated cell in Figure 5 shows a compromised cell wall and a significant shrinkage. An increase in ROS levels has been shown to cause lipid peroxidation in *Phaeodactylum tricornutum* and thereby damage the algal cells.^74^ Damaged cells had broken cell membranes and a crumpled cell surface, consistent with what is seen in the cells on the surfaces of the G and GO in Figure 5, and similar to the morphology of the UV-irradiated cell. Furthermore, the trend of the cell structures starting to deform from the extremes or edges of the cells is also consistent with the literature on the impact of oxidative stress on *Phaeodactylum tricornutum*.^75^ The decrease in cell size observed for the cells on the nanocomposite surfaces is also consistent with the physical disruption of the membrane.^76^ Along with the increase in the cell death seen in the live/dead fluorescence investigations, there is clear evidence that the G nanomaterials, particularly G in the early fouling stages, exhibit biocidal properties and cause cell death, compromising the cell membranes.

The results of the ROS quantification indicate minimal formation of hydroxyl radicals in the G and GO nanocomposite coatings with fluorescence intensities only slightly above the 3CCA control. This suggests that the oxidative stress caused by ROS generation is not a primary antifouling mechanism in this system. However, the fluorescence intensity from a sample of pure GO, which was not contained in an epoxy matrix, was significantly higher than that of the nanocomposites. Thus, there is potential for ROS generation from the nanoparticles, especially locally in the coating, on a microscopic level. However, they are not visible on a larger scale; the amounts produced are insufficient for detection using the indirect probe method with the 3CCA molecules. However, the fact that pure GO showed significant levels of hydroxyl radical generation shows that we cannot dismiss the possibility that the nanocomposite surfaces locally generate ROS that could cause oxidative stress to cells.

The spacing observed in the diffraction pattern in TEM electron diffraction of the G composite indicates that we are examining a multilayer structure rather than monolayer G. Although there is some variation in the spacing, the number of layers present likely varies, ranging from a few layers of G to structures approaching graphite. The presence of diffraction spots with a spacing of [2–10] confirms the existence of G, as this interplanar spacing is characteristic of the material. Overall, electron diffraction investigations show that the coatings contain G sheets with a distribution of layer counts. The diffraction patterns show a distribution of orientations within the material, and at times, several patterns overlap. This randomness likely results from the clustering of G. The varied orientation of the G sheets indicates that some may be protruding from the surface with their edges exposed, while others may have a flatter orientation and can cause phospholipid extraction from the cell membrane.

Since the nanocutting is primarily present in smaller sheets,^52^ having multilayers likely decreases the effect of nanocutting in our composites. G sheets have different orientations in the coating (Supporting Information), supporting electron transfer-mediated oxidative stress and phospholipid disruption hypotheses. The AF mechanism of the G sheet is likely a combination of several effects occurring sequentially or at the same time. Our results conclude that both materials’ main AF mechanisms are contact-mediated killing, not ROS-mediated oxidative stress. The contact-mediated AF mechanism could be from both (i) perpendicular sheets cutting the membrane and causing oxidative stress or (ii) parallel sheets pulling on the phospholipid molecules and causing lipid extraction.

The surface energies of all the coatings are at about 60 mN/m to 80 mN/m, significantly exceeding the range associated with fouling release strategies, which is between 20 mN/m and 30 mN/m.^69,70^ In the absence of self-cleaning mechanisms such as self-polishing or fouling release coatings, or without mechanical cleaning, the attached cells will continue to accumulate on the surfaces. It is therefore important to consider a foulant removal strategy for coatings with contact-killing antifouling mechanisms. Initial attachment is reduced upon the addition of G and GO, as seen from the results in Table 1, as the AF efficacy of G and GO significantly reduces foulant accumulation in the early stages. However, while dead cells may not proliferate as rapidly as live cells, they will still accumulate over time if not removed. In fact, research has shown that diatoms preferentially attach to or near nanoparticles in such composite coatings, likely due to attractive interactions, including electrostatic attraction between cationic nanoparticles and the negatively charged cell membranes of the diatoms.^64^

Table 2 summarizes the suggested antifouling mechanisms based on the findings of this work and the related literature.

**Table 2:** Suggested Antifouling Mechanisms of G and GO Fillers in Polymer Composites Based on This Work

Filler	Contact-mediated killing	ROS-mediated killing	Suggested AF mechanism based on findings and literature
G	Primary	Inconclusive	Phospholipid extraction
GO	Primary	Secondary	Phospholipid extraction

## Conclusions

In this paper, we have investigated the antifouling performance of G and GO epoxy composite coatings and some of the proposed antifouling mechanisms of these. G-epoxy and GO-epoxy nanocomposites demonstrate improved antifouling performance compared with pure epoxy coatings, significantly reducing diatom adhesion over time. Compared to the uncoated substrate, the epoxy-coated sample showed only 37% and 29% relative surface coverage after exposure to the mixed culture and monoculture, respectively. G and GO samples significantly improved this with 9% and 5% for G, and 8% and 4% for GO, respectively. Surface energy analysis revealed that all the coatings possess a higher surface energy of about 60–80 mN/m, significantly higher than the range for which the fouling release mechanism is effective. Quantification of ROS showed minimal generation of hydroxyl radicals from the coatings, suggesting that oxidative stress may not be the main antimicrobial pathway, although locally, at a GO agglomerate, the ROS generation could be higher, as indicated from the GO paste. Structural characterization indicated that G exists as multilayered sheets that are randomly oriented within the epoxy matrix. Consequently, some G sheets may extend out from the coating surface, exposing sharp edges. However, the presence of many-layer G rather than monolayer suggests that mechanical damage of the membrane via phospholipid extraction and oxidative stress are significant contributors to the antifouling activity, rather than nanoknife membrane penetration. Microscopy of the cells showed membrane disruption as the main cause of death, primarily by contact-mediated membrane disruption. Removal of the dead foulants remains a priority in order to take advantage of the biocidal properties of G and GO as fillers in composite coatings and their potential for less toxic marine AF coatings.

## Supplementary Material



## Notes

Additional results from experimental work, such as the contact angle and the surface free energy of the coated surfaces, fluorescence intensity measurements of the serial dilution of hydrogen peroxide exposed to the 3CCA probe molecules, and a selection of scanning electron diffraction patterns for G particles, are found in the Supporting Information (PDF)
