# Supplementary material for: The Antifouling Mechanism and Efficacy of Graphene Nanomaterials in Composite Coatings against Marine Diatoms
Source: ACS Omega. 2025 Nov 25;10(48):59478–88. doi: 10.1021/acsomega.5c09053 (PMC12771422; doi:10.1021/acsomega.5c09053)
Supplement: Supplementary file 1 [file ao5c09053_si_001.pdf]

Supplementary Information

The antifouling efficacy and mechanism of graphene nanomaterials in composite coatings against marine diatoms

Michael R. Kelly,* Andreas Erbe, Ingrid G. Hallsteinsen, Hilde L. Lein

Department of Materials Science and Engineering, Norwegian University of Science and Technology, NTNU, Sem Sælands vei 12, 7034 Trondheim, Norway

E-mail: michael.r.kelly@ntnu.no

Table S1: Measured contact angles for water and diiodomethane, together with calculated surface free energies (SFE), for all coated samples. Data represent mean ± standard deviation from three independent replicates.

Sample	Contact angle [°]		Surface free energy [mN/m]
	Water	Diiodomethane	
Epoxy	74 ± 2	47 ± 4	63 ± 3
Graphene	75 ± 1	37 ± 2	65 ± 1
Graphene oxide	67 ± 2	37 ± 1	74 ± 2

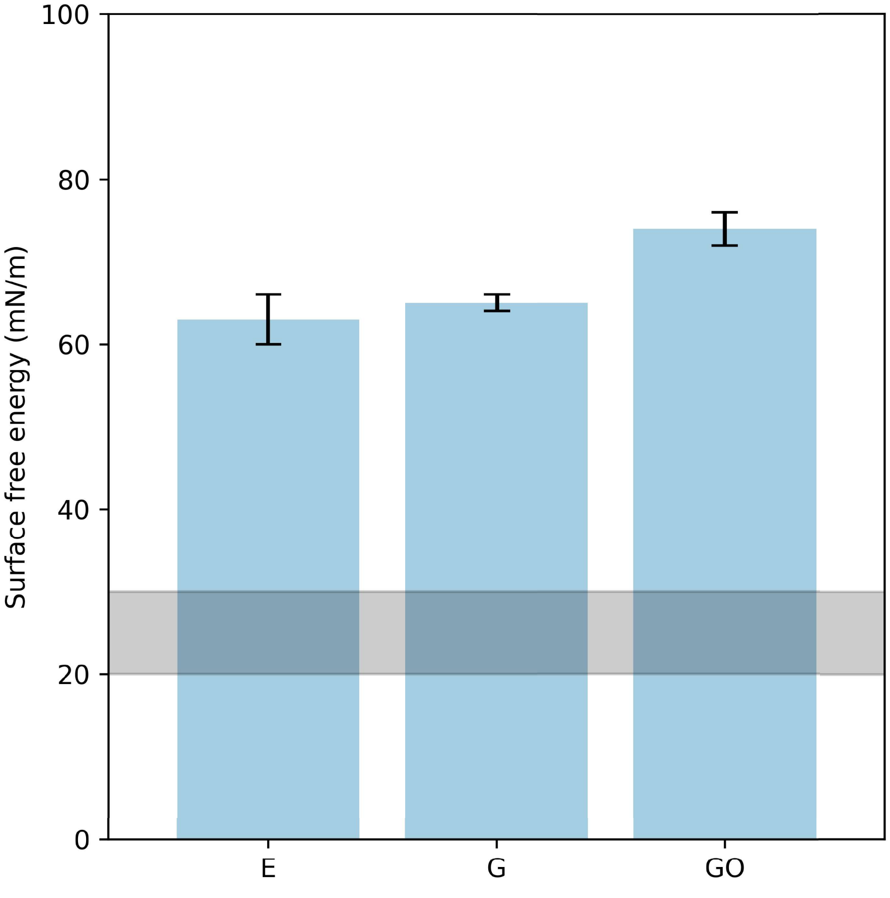

Figure S1: The surface free energy of the epoxy and composite surfaces. E = epoxy. G and GO = nanocomposite coatings with G and GO, respectively. Data represent mean ± standard deviation from three independent replicates. The grayed out area of interest is the region associated with fouling release.[69,70]

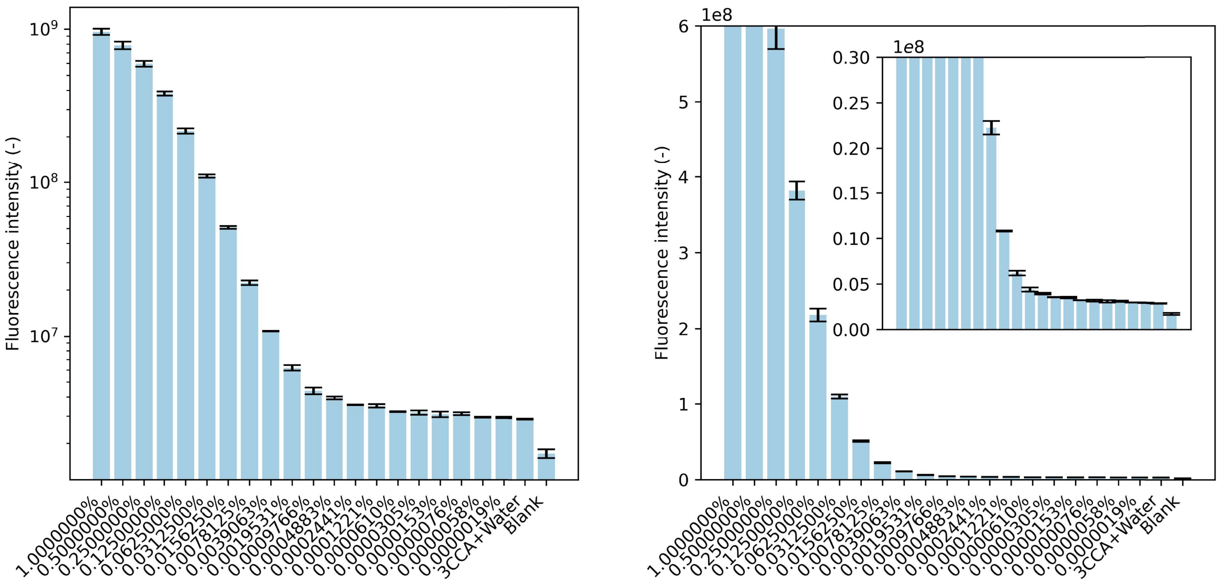

(a) (b)

Figure S2: The fluorescent intensity of the 7-hydroxycoumarin-3-carboxylic acid (HO-3CCA) probe molecule in samples exposed to hydrogen peroxide with decreasing concentration, where (a) shows in logarithmic scale, and (b) includes a zoomed inset figure with area of interest.

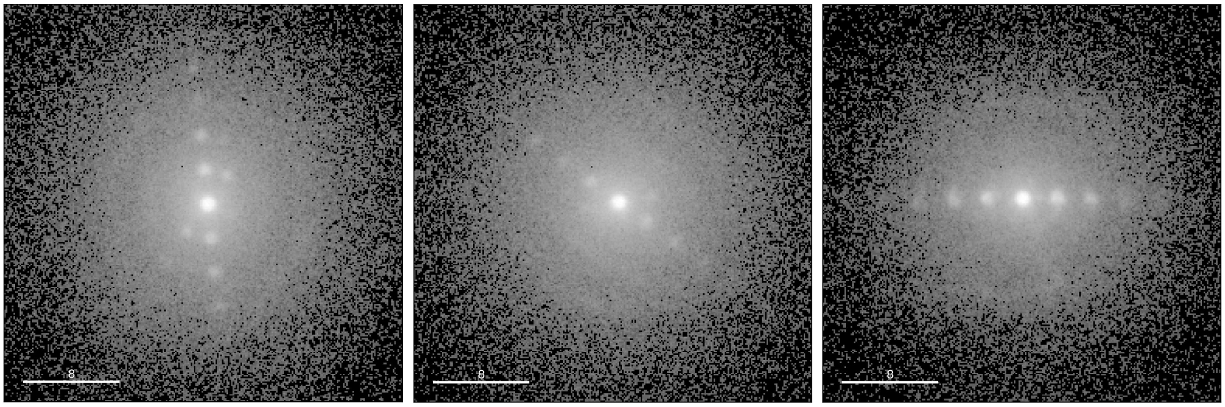

(a) (b) (c)

Figure S3: Multiple scanning electron diffraction patterns acquired from different G agglomerates, showing consistent diffraction spots indicative of G crystallinity with different orientations in the coating. Scale bars show nm^{-1}.
